# Risk-Dependent Conditional Survival and Failure Hazard After Radiotherapy for Early-Stage Extranodal Natural Killer/T-Cell Lymphoma

**DOI:** 10.1001/jamanetworkopen.2019.0194

**Published:** 2019-03-01

**Authors:** Xin Liu, Tao Wu, Su-Yu Zhu, Mei Shi, Hang Su, Ying Wang, Xia He, Li-Ming Xu, Zhi-Yong Yuan, Li-Ling Zhang, Gang Wu, Bao-Lin Qu, Li-Ting Qian, Xiao-Rong Hou, Fu-Quan Zhang, Yu-Jing Zhang, Yuan Zhu, Jian-Zhong Cao, Sheng-Min Lan, Jun-Xin Wu, Shu-Nan Qi, Yong Yang, Ye-Xiong Li

**Affiliations:** 1 State Key Laboratory of Molecular Oncology, Beijing, People’s Republic of China; 2 Department of Radiation Oncology, National Cancer Center and Cancer Hospital, Chinese Academy of Medical Sciences and Peking Union Medical College, Beijing, People’s Republic of China; 3 Center for Cancer Precision Medicine, Chinese Academy of Medical Sciences and Peking Union Medical College, Beijing, People’s Republic of China; 4 National Institute of Biological Sciences, Collaborative Innovation Center for Cancer Medicine, Beijing, People’s Republic of China; 5 Affiliated Hospital of Guizhou Medical University, Guizhou Cancer Hospital, Guiyang, Guizhou, People’s Republic of China; 6 Hunan Cancer Hospital and the Affiliated Cancer Hospital of Xiangya School of Medicine, Changsha, Hunan, People’s Republic of China; 7 Xijing Hospital, Fourth Military Medical University, Xi'an, Shaanxi, People’s Republic of China; 8 307 Hospital, Academy of Military Medical Science, Beijing, People’s Republic of China; 9 Chongqing Cancer Hospital & Cancer Institute, Chongqing, People’s Republic of China; 10 Jiangsu Cancer Hospital & Jiangsu Institute of Cancer Research, Nanjing, Jiangsu, People’s Republic of China; 11 Tianjin Medical University Cancer Institute & Hospital, Key Laboratory of Cancer Prevention and Therapy, National Clinical Research Center for Cancer, Tianjin, People’s Republic of China; 12 Union Hospital, Tongji Medical College, Huazhong University of Science and Technology, Wuhan, Hubei, People’s Republic of China; 13 The General Hospital of Chinese People's Liberation Army, Beijing, People’s Republic of China; 14 The Affiliated Provincial Hospital of Anhui Medical University, Hefei, Anhui, People’s Republic of China; 15 Peking Union Medical College Hospital, Chinese Academy of Medical Sciences and Peking Union Medical College, Beijing, People’s Republic of China; 16 Sun Yat-sen University Cancer Center, Guangzhou, Guangdong, People’s Republic of China; 17 State Key Laboratory of Oncology in South China, Guangzhou, Guangdong, People’s Republic of China; 18 Collaborative Innovation Center for Cancer Medicine, Guangzhou, Guangdong, People’s Republic of China; 19 Zhejiang Cancer Hospital, Hangzhou, Zhejiang, People’s Republic of China; 20 Shanxi Cancer Hospital and the Affiliated Cancer Hospital of Shanxi Medical University, Taiyuan, Shanxi, People’s Republic of China; 21 Fujian Provincial Cancer Hospital, Fuzhou, Fujian, People’s Republic of China

## Abstract

**Importance:**

Prognosis of early-stage extranodal natural killer/T-cell lymphoma (NKTCL) is usually estimated and stratified at diagnosis, but how the prognosis actually evolves over time for patients who survived after curative treatment is unknown.

**Objective:**

To assess conditional survival and failure hazard over time based on risk categories, previous survival, and treatment.

**Design, Setting, and Participants:**

This retrospective cohort study reviewed the clinical data of 2015 patients with early-stage NKTCL treated with radiotherapy identified from the China Lymphoma Collaborative Group multicenter database between January 1, 2000, and December 31, 2015. Patients were stratified into low-, intermediate- and high-risk groups according to a previously established prognostic model. Median follow-up was 61 months for surviving patients. Data analysis was performed from December 1, 2017, to January 30, 2018.

**Exposures:**

All patients received radiotherapy with or without chemotherapy.

**Main Outcomes and Measures:**

Conditional survival defined as the survival probability, given patients have survived for a defined time, and annual hazard rates defined as yearly event rate.

**Results:**

A total of 2015 patients were included in the study (mean [SD] age, 43.3 [14.6] years; 1414 [70.2%] male); 1628 patients (80.8%) received radiotherapy with chemotherapy, and 387 (19.2%) received radiotherapy without chemotherapy. The 5-year survival rates increased from 69.1% (95% CI, 66.6%-71.4%) at treatment to 85.3% (95% CI, 81.7%-88.2%) at year 3 for conditional overall survival and from 60.9% (95% CI, 58.3%-63.3%) at treatment to 84.4% (95% CI, 80.6%-87.6%) at year 3 for conditional failure-free survival. The annual hazards decreased from 13.7% (95% CI, 13.0%-14.3%) for death and 22.1% (95% CI, 21.0%-23.1%) for failure at treatment to less than 5% after 3 years (death: range, 0%-3.9% [95% CI, 3.7%-4.2%]; failure: 1.2% [95% CI, 1.0%-1.4%] to 4.2% [95% CI 3.9%-4.6%]). Intermediate-risk (11.4% [95% CI, 10.5%-12.3%]) and high-risk (21.6% [95% CI, 20.0%-23.2%]) patients had initially higher but significantly decreased death hazards after 3 years (<6%, range: 0%-5.9% [95% CI, 5.2%-6.7%]), whereas low-risk patients maintained a constantly lower death hazard of less than 5% (range, 0%-4.8%; 95% CI, 4.4%-5.3%). In high-risk patients, radiotherapy combined with non–anthracycline-based regimens were associated with higher conditional overall survival before year 3 compared with anthracycline-based regimens (hazard ratio [HR] for death, 1.49; 95% CI, 1.13-1.95; *P* = .004 at treatment; HR, 1.60; 95% CI, 1.07-2.39; *P* = .02 at 1 year; and HR, 1.77; 95% CI, 0.94-3.33; *P* = .07 at 2 years) or radiotherapy alone (HR, 2.42; 95% CI, 1.73-3.39; *P* < .001 at treatment; HR, 1.82; 95% CI, 1.05-3.17; *P* = .03 at 1 year; and HR, 2.69; 95% CI, 1.23-5.90; *P* = .01 at 2 years).

**Conclusions and Relevance:**

The survival probability increased and the hazards of failure decreased in a risk-dependent manner among patients with early NKTCL after radiotherapy. These dynamic data appear to provide accurate information on disease processes and continual survival expectations and may help researchers design additional prospective clinical trials and formulate risk-adapted therapies and surveillance strategies.

## Key Points

**Question:**

How do survival probabilities change over time for patients with early-stage extranodal natural killer/T-cell lymphoma after radiotherapy?

**Findings:**

In this cohort study of 2015 patients in China with early-stage natural killer/T-cell lymphoma, survival probabilities increased and failure hazard decreased over time in a risk-dependent manner after radiotherapy. Patients with an initially higher risk and constantly lower risk were identified.

**Meaning:**

The posttreatment information on disease processes may permit risk-adapted therapy, follow-up, and counseling.

## Teaser

This cohort study assesses changes in survival probabilities and failure hazard after radiotherapy in adult patients with early-stage extranodal natural killer/T-cell lymphoma based on risk categories, previous survival, and treatment.

## Introduction

Extranodal nasal-type natural killer/T-cell lymphoma (NKTCL) is a distinct entity in the World Health Organization classification.^1^ Although rare globally, this disease has a geographic predilection in East Asia and South America.^2,3,4^ Most cases originate from the upper aerodigestive tract and present as early-stage disease.^5,6,7,8^ In the past decade, non–anthracycline-based chemotherapy has improved outcomes in NKTCL.^8,9,10,11^ However, radiotherapy is still the backbone of curative intent for early-stage NKTCL, even in the modern era of chemotherapy.^12,13,14,15,16,17,18,19,20^ The efficacy of novel chemotherapy regimens in addition to radiotherapy remains to be determined for early-stage NKTCL.^10,18^

Benchmark measures of prognosis, such as 5-year cumulative statistics, are widely used to assess disease control and make general comparisons. Because of disease heterogeneity, risk stratification is used to assess prognosis for patients with NKTCL at initial diagnosis.^21,22,23^ We recently developed a prognostic nomogram model that incorporated 5 risk factors of NKTCL^22^ and demonstrated significantly different prognoses in patients with early-stage disease with and without adverse factors.^18^ However, current survival estimates and scoring models assess prognosis in a static manner at the single time point of diagnosis and do not provide accurate posttreatment information.^21,22,23^ The subsequent survival or annual hazard change over time in various risk groups of patients with early-stage NKTCL receiving radiotherapy is not fully understood. The uncertainty of prognosis for patients who have survived for several years since treatment can potentially affect clinical decision making and patients’ quality of life.

Time-dependent statistics reflect real-time changes in survival or risk at a given time point. Conditional survival and annual hazard correspondingly convey the survival probability and yearly event rate,^24,25^ given patients have survived for a defined time. These dynamic methods have been applied to various malignant tumors to assist physicians when making treatment decisions or establishing optimal surveillance schedules^26,27,28,29,30,31^ but have never been established for NKTCL. Using the updated multicenter database from the China Lymphoma Collaborative Group (CLCG), we identified patients with early-stage NKTCL treated with radiotherapy and assessed conditional survival and failure hazard in the entire cohort and for various risk categories. These data could be applied to better understand the disease process, inform patients of their updated prognosis, and formulate risk-adjusted therapy and surveillance strategies.

## Methods

### Patients and Treatment

We systematically reviewed the updated CLCG database on patients with NKTCL treated at 16 Chinese institutions between January 1, 2000, and December 31, 2015. Data analysis was performed from December 1, 2017, to January 30, 2018. A total of 2015 patients were eligible according to the following criteria: newly diagnosed NKTCL, Ann Arbor stage I to II disease, and definitive treatment with radiotherapy. Patients treated with chemotherapy alone were excluded, as chemotherapy alone is no longer considered a treatment option for early-stage NKTCL because of its poor outcome.^5,18^ As described previously,^22^ primary tumor invasion (PTI) was defined as the presence of primary disease that extended into neighboring structures or the involvement of multiple (≥2), contiguous primary sites. We followed the Strengthening the Reporting of Observational Studies in Epidemiology (STROBE) reporting guideline. The study protocol was approved by the local ethics committee from the National Cancer Center/Cancer Hospital, Chinese Academy of Medical Sciences and Peking Union Medical College. The institutional review boards approved the study protocol and waived the need for informed consent because patient data were deidentified in the data set.

### External Validation of Conditional Survival and Annual Death Hazard

To externally validate our findings, we searched PubMed and Medline using the terms *NK/T-cell lymphoma* and *nasal lymphoma*; 3 authors (X.L., Y.Y., and Y.-X.L.) read all the related publications. Eligible studies had to include Kaplan-Meier survival curves with at least 7 years of data (for available analysis of conditional survival) in patients with early-stage NKTCL treated with radiotherapy. To avoid selection bias attributable to the small number of sampled individuals, cohorts of fewer than 30 patients were excluded. We also included patients with early-stage disease who received radiotherapy in the Surveillance, Epidemiology, and End Results (SEER) database between 1992 and 2013. A total of 2315 patients from 22 cohorts were included, and of these, 306 patients were derived from the SEER database (eTable 1 in the Supplement).^4,5,8,13,14,15,32,33,34,35,36,37,38,39,40,41,42^

### Risk-Dependent Conditional Survival and Annual Hazards Over Time

We determined whether the conditional survival and annual hazards varied among the risk groups. Consistent with a previous study,^22^ the multivariable analysis showed that age older than 60 years, stage II disease, Eastern Cooperative Oncology Group (ECOG) score of 2 or higher, elevated lactate dehydrogenase (LDH) level, and the presence of PTI were independent risk factors for overall survival (OS) in early-stage NKTCL. Accordingly, we stratified patients with early-stage disease into 3 risk groups at treatment: low (no risk factors), intermediate (1 risk factor), and high (≥2 risk factors).

### Statistical Analysis

Overall survival (OS) was defined as time from the date of treatment to death from any cause. Failure-free survival (FFS) was defined as time from the date of treatment to disease progression, relapse, or death. Conditional overall survival (COS) was defined as the probability of surviving an additional number of years given that the patient has already survived a certain number of years. For example, the 5-year COS at 3 years was that the conditional probability of surviving an additional 5 years (ie, surviving to 8 years since treatment) given that the patient has survived 3 years. With extensions of the concept of conditional survival, conditional failure-free survival (CFFS) was defined as the probability of FFS with an additional number of years given that the patient has survived a certain number of years without failure.

Survival rates were estimated using the Kaplan-Meier method and compared using the log-rank test. Cox proportional hazards regression analyses used OS as the dependent variable to identify potential independent clinical prognostic factors. According to previous studies,^21,22,23^ these factors included sex, age (60 years as the cutoff), ECOG performance status, Ann Arbor stage, PTI, B symptoms (unexplained temperature >38°C, drenching night sweats, or weight loss >10% of body weight within 6 months of diagnosis), LDH level, regional lymph node involvement, and primary disease site. *P* < .05 (2-sided) was considered to be statistically significant. In published data, survival rates at different year points were extracted by using Engauge Digitizer software from Kaplan-Meier survival curve graphs, and the COS and CFFS were computed by using the standard definition of a conditional probability in statistics.^24^ Annual hazards were calculated as the annual number of events divided by total follow-up time accumulated by the patients at risk in that year. Hazard rate curves were smoothed by applying an Epanechnikov kernel.^43^ All statistical analyses were performed using SPSS, version 20.0 (IBM Inc) and R, version 3.2.1 (R Foundation for Statistical Computing).

## Results

### Cohort Characteristics

A total of 2015 patients were included in the study (mean [SD] age, 43.3 [14.6] years; 1414 [70.2%] male). The clinical characteristics of the cohort are presented in eTable 2 in the Supplement. The male to female ratio was 2.4:1. A total of 1754 patients (87.0%) were 60 years or younger, 1383 (68.6%) had stage I disease, 1921 (95.3%) had a good ECOG performance status, and 1464 (72.7%) had a normal LDH level. A PTI was present in 1088 patients (54.0%).

All patients received radiotherapy with (1628 [80.8%]) or without (387 [19.2%]) chemotherapy. Radiotherapy included an extended involved-site field at a median dose of 52 Gy. Of patients receiving chemotherapy, 827 (50.8%) received non–anthracycline-based regimens (new regimen), including asparaginase-based (585 [35.9%]), gemcitabine-based (118 [7.2%]), or platinum-based (124 [7.6%]) regimens. The remaining 782 patients (48.0%) received anthracycline-based regimens (old regimen), such as cyclophosphamide, doxorubicin, vincristine, and prednisone or similar regimens.

### Conditional Survival and Annual Hazards Over Time for the Entire Cohort

**Figure 1.**
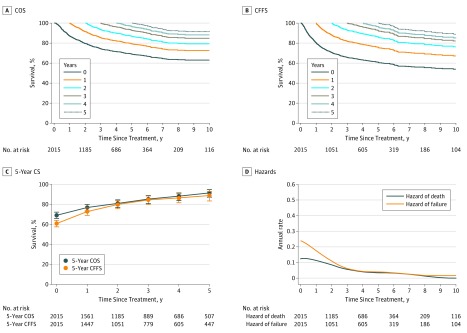
Conditional Survival and Annual Hazards of Death and Failure for Patients With Early-Stage Natural Killer/T-Cell Lymphoma Treated With Radiotherapy A, Conditional overall survival (COS) curves as a function of the number of years survived since treatment. B, Conditional failure-free survival (CFFS) curves as a function of the number of failure-free years since treatment. The colors of the lines are assigned in the order of years survived or failure-free years since treatment, from year 0 to 5. C, Five-year conditional survival (CS) probability as a function of the number of years survived or failure-free years survived since treatment. Error bars denote 95% CIs. D, Smoothed hazard plots for annual rate of death and failure by time after treatment.

With a median follow-up of 61 months for surviving patients, the 5-year survival rates at treatment were 69.1% (95% CI, 66.6%-71.4%) for OS and 60.9% (95% CI, 58.3%-63.3%) for FFS. The survival probabilities for COS and CFFS increased with each additional year. The 5-year COS increased to 76.9% (95% CI, 74.1%-79.4%) for 1-year survivorship, 81.0% (95% CI, 77.7%-83.8%) for 2-year survivorship, 85.3% (95% CI, 81.7%-88.2%) for 3-year survivorship, 88.2% (95% CI, 84.6%-91.1%) for 4-year survivorship, and 91.4% (95% CI, 87.9%-94.0%) for 5-year survivorship (Figure 1A). Similarly, the 5-year CFFS increased to 72.8% (95% CI, 69.7%-75.6%) for 1-year survivorship, 79.9% (95% CI, 76.4%-83.0%) for 2-year survivorship, 84.4% (95% CI, 80.6%-87.6%) for 3-year survivorship, 86.5% (95% CI, 82.3%-89.7%) for 4-year survivorship, and 88.8% (95% CI, 84.1%-92.1%) for 5-year survivorship (Figure 1B). Five-year COS and CFFS had the greatest increases in the first 3 years after treatment and then increased slightly in years 4 and 5 (Figure 1C).

Smoothed hazard plots illustrated the dynamics of the annual hazards of death and failure (Figure 1D) and provided more detailed information on instantaneous risk than Kaplan-Meier curves. The annual death hazard of 13.7% (95% CI, 13.0%-14.3%) and failure hazard of 22.1% (95% CI, 21.0%-23.1%) in the first year were highest, but the hazards decreased in the first 3 years. From year 4 onward, the annual death and failure hazards decreased to less than 5% (death: range, 0%-3.9% [95% CI, 3.7%-4.2%]; failure: range, 1.2% [95% CI, 1.0%-1.4%] to 4.2% [95% CI, 3.9%-4.6%]) (eTable 3 in the Supplement).

### External Validation of Conditional Survival and Annual Death Hazard

**Figure 2.**
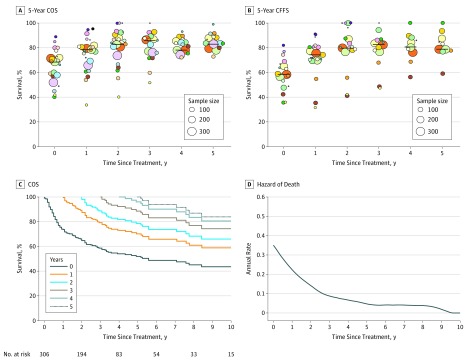
External Validation of Conditional Survival Among Patients With Early-Stage Natural Killer/T-Cell Lymphoma (NKTCL) Undergoing Radiotherapy A and B, The 5-year conditional overall survival (COS) (A) and conditional failure-free survival (CFFS) (B) over time from the 22 cohorts of 2315 patients with early-stage NKTCL treated with radiotherapy (eTable 1 in the Supplement). The center of the bubble represents the 5-year COS and 5-year CFFS of each cohort at that year. Each color represents an individual cohort. The lines represent the median 5-year COS and 5-year CFFS of all cohorts. The figures are staggered for visual effect. C and D, The COS (C) and smoothed hazard plots (D) for annual rate of death as a function of the number of years survived since treatment for patients with early-stage NKTCL who underwent radiotherapy from the Surveillance, Epidemiology, and End Results database.

Externally validated data showed that the median 5-year OS rate of 68.5% (range, 40.3%-89.0%) was comparable to the corresponding rate of 69.1% in the cohort in our study, as was the median 5-year FFS rate of 59.0% (range, 36.0%-82.1%) and our rate of 60.9%. Conditional survival increased over time in each cohort or in the overall analysis of all cohorts (Figure 2A and B). Given a 3-year survivorship or failure-free survivorship, the median 5-year COS increased to 86.9% (range, 52.4%-100%) and the median 5-year CFFS increased to 84.3% (range, 48.2%-100%) in all studies.

Because the individual survival data were available, we conducted an independent validation from the SEER cohort. Although the 5-year OS rate was 52.0% (95% CI, 45.2%-58.3%) at treatment, the 5-year COS at year 3 increased to 77.0% (95% CI, 65.2%-85.3%) (Figure 2C). Similarly, the annual death hazard from the SEER data was high (30.3%) in the first year but decreased from 3% to 8% from year 4 onward (Figure 2D).

### Risk-Dependent Conditional Survival and Annual Hazards Over Time

**Figure 3.**
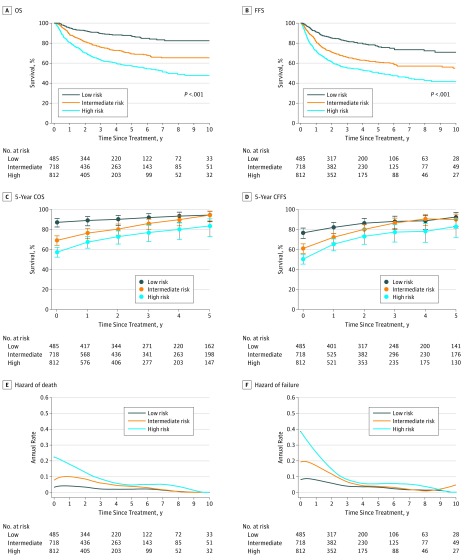
Kaplan-Meier Survival Curves, Conditional Survival, and Hazards Stratified by Risk Groups A and B, Kaplan-Meier curves of overall survival (OS) (A) and failure-free survival (FFS) (B) at treatment in low-, intermediate-, and high-risk patients. C and D, The 5-year conditional overall survival (COS) (C) and conditional failure-free survival (CFFS) (D) over time in low-, intermediate-, and high-risk patients. Lines connecting the conditional survival for each time are linear connectors between estimates. Error bars denote 95% CIs. E and F, Smoothed hazard plots for death (E) and failure (F) over time in low-, intermediate-, and high-risk patients.

Each risk group achieved significantly different 5-year OS and FFS after treatment, with rates of 87.2% (95% CI, 83.2%-90.3%) for OS and 76.7% (95% CI, 71.8%-80.8%) for FFS in the low-risk group, 69.3% (95% CI, 65.0%-73.1%) for OS and 61.1% (95% CI, 56.8%-65.0%) for FFS in the intermediate-risk group, and 57.5% (95% CI, 53.2%-61.5%) for OS and 50.4% (95% CI, 46.2%-54.5%) for FFS in the high-risk groups (*P* < .001 for all comparisons), suggesting excellent discrimination (Figure 3A and B).

Five-year conditional survival probabilities increased mainly in high- and intermediate-risk groups over time but remained excellent in low-risk patients (Figure 3C and D). Intermediate-risk patients who survived or were failure free beyond 3 years achieved comparable 5-year COS and CFFS with low-risk patients (eTable 4 and eTable 5 in the Supplement). However, high-risk patients constantly maintained inferior COS rates compared with low-risk patients at any time point (eFigure and eTable 4 in the Supplement).

Low-risk patients had constantly lower risk: annual hazards were 4.8% (95% CI, 4.4%-5.3%) for death and 9.1% (95% CI, 8.3%-10.0%) for failure in the first year, which then decreased to less than 3% (range, 0%-2.9%; 95% CI, 1.5%-2.2%) for death and less than 4.2% for failure (range, 1.2% [95% CI, 1.0%-1.4%] to 4.2% [95% CI, 3.9%-4.6%]) after 3 years (eTable 3 in the Supplement). In contrast, high-risk and intermediate-risk patients had an initially higher risk. In the first year, annual death hazards were 21.6% (95% CI, 20.0%-23.2%) for high-risk patients and 11.4% (95% CI, 10.5%-12.3%) for intermediate-risk patients, whereas annual failure hazards were 32.7% (95% CI, 30.2%-35.2%) for high-risk patients and 20.1% (95% CI, 18.5%-21.6%) for intermediate-risk patients. However, the death and failure hazards decreased to less than 6% for death (range, 0%-5.9%; 95% CI, 5.2%-6.7%) and failure (range, 0%-5.9%; 95% CI, 5.0%-6.8%) after 3 years (Figure 3E and F and eTable 3 in the Supplement).

### Risk-Dependent Survival Benefit and Hazards According to Treatment

**Figure 4.**
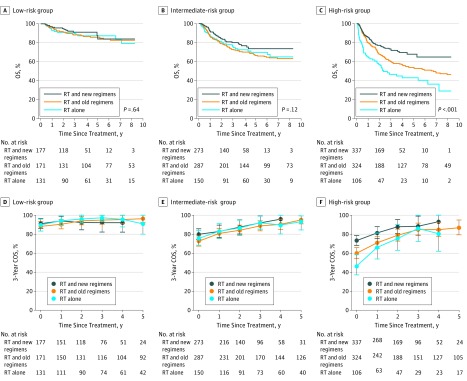
Kaplan-Meier Survival Curves and Conditional Survival Stratified by Treatment in Each Risk Group A-C, Kaplan-Meier curves of overall survival (OS) for radiotherapy (RT) and non–anthracycline-based (new) regimens, RT and anthracycline-based (old) regimens, and RT alone in low-risk (A), intermediate-risk (B), and high-risk (C) patients. D-F, The 3-year conditional overall survival (COS) for RT and new regimens, RT and old regimens, and RT alone in low-risk (D), intermediate-risk (E), and high-risk (F) patients. Lines connecting the conditional survival for each time are linear connectors between estimates. Error bars denote 95% CIs.

We then determined whether the combination of chemotherapy and radiotherapy was associated with the evolution of survival probabilities and hazards. In the overall cohort, the 5-year OS (74.5%) and FFS (65.9%) for radiotherapy and a new chemotherapy regimen were better than for radiotherapy and an old chemotherapy regimen (65.2% for OS; HR for death, 0.70; 95% CI, 0.57-0.85, *P* < .001; 55.1% for FFS; HR for failure or death, 0.70; 95% CI, 0.57-0.85, *P* < .001) or radiotherapy alone (68.3% for OS; HR for death, 0.70; 95% CI, 0.55-0.90; *P* = .005; 62.9% for FFS; HR for failure or death, 0.82; 95%, CI 0.66-1.02; *P* = .08). After risk stratification, no difference was found in OS among the 3 treatment groups in the low-risk (Figure 4A and eTable 6 in the Supplement) or intermediate-risk patients (Figure 4B and eTable 7 in the Supplement). However, the 5-year OS for radiotherapy and a new chemotherapy regimen (67.8%) was significantly better than that for radiotherapy and an old chemotherapy regimen (53.8%; HR for death, 0.67; 95% CI, 0.51-0.88; *P* = .004) or radiotherapy alone (43.0%; HR for death, 0.41; 95% CI, 0.30-0.58; *P* < .001) in high-risk patients (Figure 4C).

**Figure 5.**
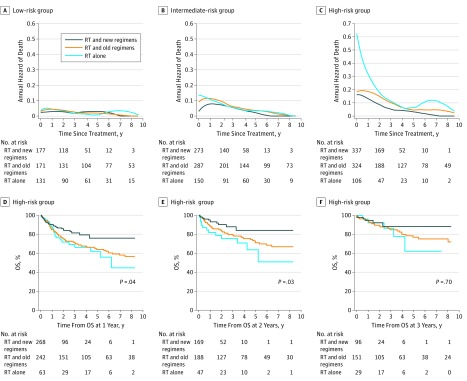
Annual Death Hazard Stratified by Treatment in Each Risk Group and Conditional Overall Survival in High-Risk Patients A-C, Smoothed hazard plots for death over time in low-risk (A), intermediate-risk (B), and high-risk (C) patients. D-F, Kaplan-Meier curves of overall survival (OS) between radiotherapy (RT) and non–anthracycline-based (new) regimens, RT and anthracycline-based (old) regimens, and RT alone in high-risk patients who were surviving at 1 year (D), 2 years (E), and 3 years (F).

No difference in COS was found among the 3 treatment groups in low- or intermediate-risk patients for any landmark analysis (Figure 4D and E; eTable 6 and eTable 7 in the Supplement). However, a significantly greater COS was observed for radiotherapy plus a new chemotherapy regimen compared with radiotherapy plus an old chemotherapy regimen (HR for death, 1.49; 95% CI, 1.13-1.95; *P* = .004 at treatment; HR for death, 1.60; 95% CI, 1.07-2.39; *P* = .02 at 1 year; and HR for death, 1.77; 95% CI, 0.94-3.33; *P* = .07 at 2 years) or radiotherapy alone (HR for death, 2.42; 95% CI, 1.73-3.39; *P* < .001 at treatment; HR for death, 1.82; 95% CI, 1.05-3.17; *P* = .03 at 1 year; and HR for death, 2.69; 95% CI, 1.23-5.90; *P* = .01 at 2 years) before the landmark of 3 years in high-risk patients (Figure 4F and eTable 8 in the Supplement). After patients received radiotherapy, the annual death hazard remained low in the low-risk group (Figure 5A) independent of whether the patients received additional old or new chemotherapy regimen. However, this hazard decreased over time in the intermediate- or high-risk groups (Figure 5B and C). In high-risk patients, radiotherapy with a new chemotherapy regimen was associated with an increase in COS compared with radiotherapy plus an old chemotherapy regimen (HR for death, 1.49; 95% CI, 1.13-1.95; *P* = .004 at treatment; HR for death, 1.60; 95% CI, 1.07-2.39; *P* = .02 at 1 year; and HR for death, 1.77; 95% CI, 0.94-3.33; *P* = .07 at 2 years) or radiotherapy alone (HR for death, 2.42; 95% CI, 1.73-3.39; *P* < .001 at treatment; HR for death, 1.82; 95% CI, 1.05-3.17; *P* = .03 at 1 year; and HR for death, 2.69; 95% CI, 1.23-5.90; *P* = .01 at 2 years) at the landmark of 1 year (Figure 5D) and 2 years (Figure 5E), but this benefit was not significant when patients survived up to 3 years (Figure 5F and eTable 8 in the Supplement). This finding suggested a risk-dependent dynamic change in conditional survival and annual hazards according to treatment.

## Discussion

Because of its rarity worldwide, any attempt to assess the conditional survival or annual hazards for NKTCL is challenging. In this large cohort of patients with early-stage NKTCL uniformly treated with radiotherapy from the CLCG database, survival probability increased, whereas the hazards of failure and death decreased as survival time accrued. This finding was externally validated by independent data from published studies and the SEER database. Further analysis demonstrated a risk-dependent pattern: intermediate- and high-risk patients had an initially higher but significantly decreased risk of failure and death over time, whereas low-risk patients had a constantly lower risk. Combining a new chemotherapy regimen with radiotherapy provided a survival benefit in high-risk patients but not in low- or intermediate-risk patients. Within each risk group, time-dependent survival and hazards after radiotherapy showed similar risk-dependent patterns across different treatment modalities. These findings add to the accuracy of continual prognostic estimates after treatment and provide the rationale for risk-adapted therapy and surveillance strategies in this population.

During the follow-up of patients who survived initial treatment, information on their current prognosis is important to patients and practitioners. In this study, we introduced a conditional approach to reflect the time-dependent changes in survival probability and risk of failure for early-stage NKTCL treated with radiotherapy. At treatment, the 5-year rates of 69% for OS and 61% for FFS in this cohort were comparable with contemporary outcomes for radiotherapy with or without anthracycline-based or non–anthracycline-based chemotherapy.^4,6,7,8,9,13,14,15,16,17,18,19,20,40,41,42^ Conditional survival rates increased greatly in the first 3 years and slightly thereafter. The low annual hazards of death and failure after 3 years suggest that late death or failure occur rarely after definitive radiotherapy. Despite the heterogeneity in clinical features and treatments (eg, chemotherapy regimens or radiotherapy doses or techniques), this significant time-dependent survival improvement was externally validated by a second set of analyzed data drawn from published studies and the SEER database exploring patients with early-stage NKTCL who were undergoing radiotherapy. From a dynamic standpoint, this finding suggests that a longer period of survival or remission from disease was associated with an increased probability of further survival or remission from disease. Outliving a 3-year landmark was associated with excellent long-term survival after radiotherapy for patients with early-stage NKTCL. In comparison, achieving event-free survival for 1 to 2 years is associated with low failure risk and excellent outcomes in other lymphomas primarily treated with chemotherapy.^44,45,46,47^ The survival landmark variations may reflect the heterogeneous clinical behaviors after different curative therapies. Consistent with conditional analysis for other malignant tumors in the curative context,^26,27,28,29^ improved survival and decreased hazards of failure and mortality over time highlight the long-term curability of radiotherapy for early-stage NKTCL.

Previous reports^21,22,23^ have more explicitly highlighted the importance of initial prognosis and treatment in individual NKTCL cases by using clinical features at diagnosis. However, these analyses were based on proportional hazards models and implicitly assumed a constant risk of covariates. Data on how individual prognosis actually evolves over time after treatment are lacking. In this study, after stratifying patients with early-stage disease into 3 subgroups using 5 established adverse factors (age >60 years, stage II disease, ECOG score ≥2, elevated LDH level, and the presence of PTI), conditional survival improved more clearly in the high- and intermediate-risk patients as survival time accrued. In contrast, conditional survival remained excellent in low-risk patients. High- and intermediate-risk patients were more likely to experience consistent early failure and death after primary radiotherapy (during the first 3 years). However, the annual hazards decreased over time, resulting in comparable low hazards (<5%) in the low-risk patients after 3 years. Conversely, low-risk patients maintained a lower risk of death and failure. Consequently, despite the heterogeneous prognoses for each risk group at treatment, all patients attained an equivalent favorable survival probability (80%-90%) after surviving or achieving FFS for 3 years regardless of their initial risk category. Accordingly, we introduced a new term of *constantly lower risk* to low-risk patients (with no risk factors) and *initially higher risk* to high- and intermediate-risk patients (with ≥1 risk factor) who had more hazards at treatment but reduced hazards during follow-up. Therefore, the current prognostic models based on constant risk assumptions may overestimate the risk of late failure or mortality after 3 years for initially higher-risk patients.^18,21,22,23^

Our findings suggest that addition of non–anthracycline-based chemotherapy to radiotherapy is associated with survival benefit in high-risk patients. After frontline radiotherapy (regardless of chemotherapy or chemotherapy regimens), the annual death hazard in high- and intermediate-risk patients decreased over time but remained stable in low-risk patients during follow-up. Patients in each risk group presented with a similar risk-dependent pattern of survival probability and annual hazards across different treatment strategies. This finding reinforces the necessity of risk-adapted therapy with a pivotal role of frontline radiotherapy and contributes to more individualized clinical decision making in early-stage NKTCL.^18^ If a patient is categorized as being initially at higher risk, aggressive or intensified systemic therapeutic intervention should be considered at diagnosis. However, this approach potentially overtreats the constantly lower-risk patients.

The different dynamics of annual failure hazards among risk categories support the rationale and necessity of a risk-adapted surveillance scheme because evidence-based recommendations are not yet possible for early-stage NKTCL with regard to the ideal frequency and timing of follow-up visits. Because risk of treatment failure has been reported to be an important criterion for individualizing follow-up protocols for patients with cancer,^48,49^ the frequency of surveillance can be formulated by adjusting the number of failures detected for each 100 patient visits during a follow-up interval. This approach is more evidence based than simply relying on traditional schedules. Regardless of treatment strategies, initially higher-risk patients may require more intensive surveillance during the first 3 years (eg, every 3 months) and an appropriately reduced follow-up frequency after 3 years (eg, every 6 months to annually). However, constantly lower-risk patients may require less frequent follow-up (eg, every 3 months in the first year, every 6 months in the first 3 years, and annually thereafter). Such a risk-adapted surveillance scheme may be associated with reduced anxiety, radiation exposure, and health care costs for constantly lower-risk patients, while enabling early detection of failure to provide timely additional treatment for those patients who are initially higher risk.

### Limitations

This study has several limitations. Because primary radiotherapy results in better outcomes than chemotherapy alone or even non–anthracycline-based chemotherapy in early-stage NKTCL,^5,18,20,50^ the conditional survival and hazard estimates reported here may not be generalizable to patients with advanced-stage disease or patients treated with primary chemotherapy. Future studies should focus on these subsets, although this may be difficult because of the small number of patients and the poor outcomes. In addition, the heterogeneous regimens in this study reflect the lack of consensus on the standard chemotherapy regimen for patients with early-stage NKTCL. Survival benefit and dynamic changes of survival probability and hazards in patients with early-stage disease treated with combined innovative chemotherapy and radiotherapy warrant further investigation.

## Conclusions

These findings suggest that risk-dependent changes in survival and risk of failure over time for patients with early-stage NKTCL after radiotherapy provide accurate information on disease processes and continual survival expectations. These valuable dynamic data may better enable the design of prospective clinical trials, while optimizing risk-adjusted therapy and surveillance strategies for this specific lymphoma.

## Notes

Supplement. **eTable 1.** Survival outcomes in early-stage NKTCL treated with primary radiotherapy reported in the literature **eTable 2.** Clinical characteristics of the overall cohort of patients with early-stage NKTCL treated with first-line radiotherapy **eTable 3**. Annual hazards of death and failure for patients with early-stage natural killer/T-cell lymphoma treated with radiotherapy and for patients in each risk group **eTable 4.** Five-year conditional overall survival probabilities for different risk groups over time **eTable 5.** Five-year conditional failure-free survival probabilities for different risk groups over time **eTable 6.** Three-year conditional overall survival probabilities stratified by treatment in low-risk patients **eTable 7.** Three-year conditional overall survival probabilities stratified by treatment in intermediate-risk patients **eTable 8.** Three-year conditional overall survival probabilities stratified by treatment in high-risk patients **eFigure.** Kaplan-Meier curves of overall survival stratified by risk groups
