# Supplementary material for: Risk-Dependent Conditional Survival and Failure Hazard After Radiotherapy for Early-Stage Extranodal Natural Killer/T-Cell Lymphoma
Source: JAMA Netw Open. 2019 Mar 1;2(3):e190194. doi: 10.1001/jamanetworkopen.2019.0194 (PMC6484659; doi:10.1001/jamanetworkopen.2019.0194)
Supplement: Supplement. — eTable 1. Survival outcomes in early-stage NKTCL treated with primary radiotherapy reported in the literature eTable 2. Clinical characteristics of the overall cohort of patients with early-stage NKTCL treated with first-line radiotherapy eTable 3. Annual hazards of death and failure for patients with early-stage natural killer/T-cell lymphoma treated with radiotherapy and for patients in each risk group eTable 4. Five-year conditional overall survival probabilities for different risk groups over time eTable 5. Five-year conditional failure-free survival probabilities for different risk groups over time eTable 6. Three-year conditional overall survival probabilities stratified by treatment in low-risk patients eTable 7. Three-year conditional overall survival probabilities stratified by treatment in intermediate-risk patients eTable 8. Three-year conditional overall survival probabilities stratified by treatment in high-risk patients eFigure. Kaplan-Meier curves of overall survival stratified by risk groups [file jamanetwopen-2-e190194-s001.pdf]

Supplementary Online Content

Liu X, Wu T, Zhu S-Y, et al. Risk-dependent conditional survival and failure hazard after radiotherapy for early-stage extranodal natural killer/T-cell lymphoma. *JAMA Netw Open*. 2019;2(3):e190194. doi:10.1001/jamanetworkopen.2019.0194

eTable 1. Survival outcomes in early-stage NKTCL treated with primary radiotherapy reported in the literature

eTable 2. Clinical characteristics of the overall cohort of patients with early-stage NKTCL treated with first-line radiotherapy

eTable 3. Annual hazards of death and failure for patients with early-stage natural killer/T-Cell Lymphoma treated with radiotherapy and for patients in each risk group

eTable 4. Five-year conditional overall survival probabilities for different risk groups over time

eTable 5. Five-year conditional failure-free survival probabilities for different risk groups over time

eTable 6. Three-year conditional overall survival probabilities stratified by treatment in low-risk patients

eTable 7. Three-year conditional overall survival probabilities stratified by treatment in intermediate-risk patients

eTable 8. Three-year conditional overall survival probabilities stratified by treatment in high-risk patients

eFigure. Kaplan-Meier curves of overall survival stratified by risk group**s**

This supplementary material has been provided by the authors to give readers additional information about their work.

© 2019 Liu X et al. *JAMA Network Open*.

eTable 1. Survival outcomes in early-stage NKTCL treated with primary radiotherapy reported in the literature

Author	Cohort number	Total number	Stage	Treatment	Chemotherapy regimen	Median RT dose (Gy)	5-year OS (%)	5-year PFS or DFS (%)
Cheung 2002 [35]	1	79	I-II	CMT: 61	anthracycline-based	50	40	36
Kim 2005 [36]	2	53	I-II	RT: 33, CMT: 20	CHOP 100%	45	69	NA
Li 2006 [13]	3	105	I-II	RT: 31, CMT: 71, CT: 3	CHOP 100%	50	71	59
Huang 2008 [14]†	4	82	I-II	RT: 9, CMT: 65	CHOP 100%	50	57	42
Ma 2010 [37]	5	64	I-II	RT: 23, CMT: 41	CHOP 90%	54	59	52
Li 2011 [38]	6	214	I-II	RT: 96, CMT: 118	CHOP 85%	50	72	65
Li 2012 [15]	7	87	I	RT: 87	No	50	80	69
Yamaguchi 2012 [8]	8	33	I-II	CCRT: 33	DeVIC 100%	50	73	67
Fang 2014 [39]	9	124	II	CMT: 84, RT: 30, CT: 10	CHOP 83%	50	60	48
Oh 2015 [19]	10	62	I-II	CCRT: 62	VIPD 52%, L-ASP-based 48%	40	83 (3-year)	77 (3-year)

© 2019 Liu X et al. *JAMA Network Open*.

Dong 2016 [40]	11	78	I-II	CMT: 33	DICE-L-ASP 100%	45/55	89	82
	12			RT: 45			49	49
Moon 2016[41]†	13	158	I-II	CT + RT: 61	CHOP 47%, non-anthracycline- based 53%	43.2	78	63
	14			CRT + CT:55		43.2	70	53
Huang 2017 [42]	15	44	I-II	IMRT + CT: 44	GDP		85 (3-year)	77 (3-year)
Cao 2017 [44]†	16	94	I-II	CRT: 43	CHOP	50	42	36
Yang 2017 [45]	17	37	I-II	RT ± CT: 29, RT alone 8	CHOP 55%, LVP 28, others 15%	50	76 (3-year)	64 (3-year)
Kwong 2017 [43]*	18	303	I-II	RT ± CT: 271, CT alone: 32	Non-anthracycline-based	40–52.8	NA	NA
Yamaguchi 2017[4]*	19	257	I-II	CCRT: 183, SCRT: 39, RT alone: 22, CT alone 11	Non-anthracycline-based 78% (DeVIC 66%)	50	68	56
Vargo 2017 [5]*	20	642	I-II	CMT: 161	NA	50	58	NA
	21			RT alone：65			45	NA

© 2019 Liu X et al. *JAMA Network Open*.

SEER (1992-2013)*	22	306	I-II	CMT: 218, RT alone: 88	NA	NA	52	NA

* multicenter cohort.

† patients who received chemotherapy only were excluded from this analysis.

Abbreviations: NKTCL, extranodal NK/T-cell lymphoma, nasal-type; RT, radiotherapy; CT: chemotherapy; OS, overall survival; PFS, progression-free survival; DFS, disease-free survival; NA, not available; CMT, combined modality therapy; CCRT, concurrent chemoradiotherapy; SCRT: sequential chemoradiotherapy; CHOP: cyclophosphamide, doxorubicin, vincristine and prednisolone; IMEP: ifosfamide, methotrexate, etoposide and prednisone; DeVIC: dexamethasone, etoposide, ifosfamide, and carboplatin; DEP: dexamethasone, VP-16 and cisplatin; GELOX, gemcitabine, oxaliplatin, and *L*-asparaginase; VIDP: etoposide, ifosfamide, cisplatin, and dexamethasone; L-ASP, *L*-asparaginase; GEM-based, gemcitabine-based; CEOP: cyclophosphamide, epirubicin, vincristine and prednisone; DICE: etoposide, cyclophosphamide, cisplatin, and dexamethasone; VIDL: etoposide, ifosfamide, dexamethasone, and *L*-asparaginase; GDP, gemcitabine, dexamethasone, and cisplatin; EPOCH, etoposide, doxorubicin, vincristine, cyclophosphamide, and prednisone; LVP, *L*-asparaginase, vincristine, and prednisone; DICE-L-ASP, cisplatin, ifosfamide, etoposide, dexamisone, *L*-asparaginase; SEER, the Surveillance, Epidemiology and End Results.

© 2019 Liu X et al. *JAMA Network Open*.

eTable 2. Clinical characteristics of the overall cohort of patients with early-stage NKTCL treated with first-line radiotherapy

Characteristics	Number (%)
Gender	
Male	1414 (70.2)
Female	601 (29.8)
Age (years)	
⩽ 60	1754 (87.0)
> 60	261 (13.0)
B symptoms	
No	1259 (62.5)
Yes	756 (37.5)
Stage	
I	1383 (68.6)
II	632 (31.4)
Primary tumor invasion	
No	927 (46.0)
Yes	1088 (54.0)
Elevated LDH	

© 2019 Liu X et al. *JAMA Network Open*.

No	1464 (72.7)
Yes	551 (27.3)
ECOG score	
0-1	1921 (95.3)
⩾ 2	94 (4.7)
Risk groups*	
Low (no risk factor)	485 (24.1)
Intermediate (1 risk factor)	718 (35.6)
High (⩾ 2 risk factors)	812 (40.3)

*Risk factors included: age >60 years, stage II disease, ECOG score ⩾2, elevated LDH, and the presence of primary tumor invasion.

Abbreviations: NKTCL, extranodal nasal-type NK/T-cell lymphoma; LDH, lactate dehydrogenase; ECOG, Eastern Cooperative Oncology Group.

© 2019 Liu X et al. *JAMA Network Open*.

eTable 3. Annual hazards of death and failure for patients with early-stage natural killer/T-Cell Lymphoma treated with radiotherapy

	1 year	2 year	3 years	4 years	5 years	6 years	7 years	8 years	9 years	10 years
All patients										
Annual hazards of death (%)	13.7	9.0	6.8	3.9	3.5	3.0	3.8	1.6	0.6	0
95% CI (%)	13.0-14.3	8.5-9.5	6.3-7.2	3.7-4.2	3.3-3.8	2.7-3.3	3.4-4.2	1.4-1.8	0.5-0.6	NA
Annual hazards of failure (%)	22.1	13.0	7.5	3.7	4.2	3.7	3.6	1.8	1.2	1.6
95% CI (%)	21.0-23.1	12.3-13.8	7.0-8.0	3.4-3.9	3.9-4.6	3.3-4.0	3.2-4.0	1.6-2.0	1.0-1.4	1.3-1.9
Low-risk patients										
Annual hazards of death (%)	4.8	3.1	2.9	1.6	1.1	2.9	1.9	1.2	0	0
95% CI (%)	4.4-5.3	2.8-3.5	2.6-3.3	1.4-1.8	0.9-1.2	2.4-3.4	1.5-2.2	0.9-1.4	NA	NA
Annual hazards of failure (%)	9.1	7.0	3.6	3.7	4.2	3.7	3.6	1.8	1.2	1.6
95% CI (%)	8.3-10.0	6.3-7.7	3.1-4.0	3.4-3.9	3.9-4.6	3.3-4.0	3.2-4.0	1.6-2.0	1.0-1.4	1.3-1.9
Intermediate -risk patients										
Annual hazards of death (%)	11.4	9.0	6.7	4.3	4.8	1.8	4.1	0	0	0
95% CI (%)	10.5-12.3	8.2-9.8	6.0-7.4	3.9-4.8	4.1-5.4	1.5-2.1	3.4-4.8	NA	NA	NA
Annual hazards of failure (%)	20.1	14.1	7.7	4.6	3.0	3.4	3.7	0	0	3.6
95% CI (%)	18.5-21.6	12.8-15.4	6.9-8.5	4.1-5.2	2.6-3.4	2.8-3.9	3.0-4.3	NA	NA	2.7-4.6

© 2019 Liu X et al. *JAMA Network Open*.

High-risk patients										
Annual hazards of death (%)	21.6	13.6	10.4	5.9	4.6	4.8	5.7	4.7	2.2	0
95% CI (%)	20.0-23.2	12.4-14.8	9.3-11.5	5.2-6.7	3.9-5.3	3.9-4.6	4.5-6.9	3.5-5.8	1.6-2.9	NA
Annual hazards of failure (%)	32.7	17.0	11.1	3.5	5.9	5.4	5.2	5.2	2.6	0
95% CI (%)	30.2-35.2	15.4-18.6	9.8-12.4	3.0-4.0	5.0-6.8	4.4-6.4	4.0-6.3	3.9-6.6	1.8-3.4	NA

Abbreviations: CI, confidence interval; NA, not available.

© 2019 Liu X et al. *JAMA Network Open*.

eTable 4. Five-year conditional overall survival probabilities for different risk groups over time

		Time since treatment					
Risk groups		0 year	1 year	2 years	3 years	4 years	5years
Low-risk	5-year COS	87.2	89.1	90.2	91.9	93.4	94.4
	95% CI	83.2-90.3	84.4-92.4	84.9-93.7	86.1-95.4	87.5-96.6	88.6-97.4
Intermediate-risk	5-year COS	69.3	76.5	80.5	86.1	90.0	94.4
	95% CI	65.0-73.1	71.8-80.5	75.0-84.9	80.6-90.2	84.4-93.5	89.1-97.2
High-risk	5-year COS	57.5	67.5	73.0	77.1	80.2	83.6
	95% CI	53.2-61.5	62.0-72.3	66.1-78.7	68.6-83.5	70.7-86.8	73.7-90.1
Intermediate-risk vs. low-risk	HR for death	2.41	2.42	2.21	2.15	1.95	1.37
	95% CI	1.79-3.24	1.66-3.53	1.38-3.53	1.18-3.93	0.96-4.00	0.56 to 3.20
	P	< 0.001	< 0.001	0.001	0.010	0.06	0.47
High-risk vs. low-risk	HR for death	4.01	3.66	3.31	3.15	2.93	2.58
	95% CI	3.01-5.33	2.52-5.30	2.07-5.29	1.70-5.80	1.41-6.12	1.11-5.99
	P	< 0.001	< 0.001	< 0.001	<0.001	0.003	0.02

Abbreviations: COS, conditional overall survival; HR, hazard ratio; CI, confidence interval.

© 2019 Liu X et al. *JAMA Network Open*.

eTable 5. Five-year conditional failure-free survival probabilities for different risk groups over time

		Time since treatment					
Risk groups		0 year	1 year	2 years	3 years	4 years	5years
Low-risk	5-year CFFS	76.7	82.1	86.3	88.1	88.7	92.4
	95% CI	71.8-80.8	76.8-86.4	80.4-90.6	81.4-92.5	80.7-93.5	84.0-96.5
Intermediate-risk	5-year CFFS	61.1	72.3	80.2	86.5	90.6	90.0
	95% CI	56.8-65.0	67.3-76.7	76.4-84.8	80.6-90.7	84.7-94.3	81.9-94.6
High-risk	5-year CFFS	50.4	65.4	73.2	77.4	78.2	83.0
	95% CI	46.2-54.5	59.5-70.6	65.8-79.2	68.3-84.2	67.9-85.5	72.2-89.9
Intermediate-risk vs. low-risk	HR for failure or death	1.96	1.77	1.60	1.36	1.23	1.59
	95% CI	1.55-2.47	1.30-2.42	1.05-2.44	0.81-2.28	0.66-2.27	0.74-3.43
	P	< 0.001	< 0.001	0.03	0.25	0.52	0.23
High-risk vs. low-risk	HR for failure or death	2.83	2.26	2.15	1.70	1.85	2.19
	95% CI	2.26-3.54	1.66-3.08	1.41-3.28	0.99-2.91	0.99-3.45	0.98-4.91
	P	< 0.001	< 0.001	< 0.001	0.05	0.05	0.05

Abbreviations: CFFS, conditional failure-free survival; HR, hazard ratio; CI, confidence interval.

© 2019 Liu X et al. *JAMA Network Open*.

eTable 6. Three-year conditional overall survival probabilities stratified by treatment in low-risk patients

		Time since treatment					
Treatment		0 year	1 year	2 years	3 years	4 years	5years
RT and new CT regimen	3-year COS	91.1	93.9	92.3	92.2	92.2	NA
	95% CI	86.3-96.2	89.6-98.5	84.6-100	82.3-100	82.3-100	NA
RT and old CT regimen	3-year COS	88.3	90.5	94.1	95.4	95.4	96.3
	95% CI	83.3-93.5	95.7-95.6	89.9-98.5	91.0-99.9	91.0-99.9	92.4-100
RT alone	3-year COS	89.8	94.1	96.0	97.3	95.7	90.9
	95% CI	84.4-95.5	89.2-99.3	91.7-100	93.6-100	87.7-100	79.6-100
	P	0.64	0.56	0.83	0.97	0.76	0.99

Abbreviations: RT, radiotherapy; CT: chemotherapy; COS, conditional overall survival; HR, hazard ratio; CI, confidence interval; NA, not available.

© 2019 Liu X et al. *JAMA Network Open*.

eTable 7. Three-year conditional overall survival probabilities stratified by treatment in intermediate-risk patients

		Time since treatment					
Treatment		0 year	1 year	2 years	3 years	4 years	5years
RT and new CT regimen	3-year COS	80.0	83.0	87.5	92.0	95.9	NA
	95% CI	74.5-85.8	76.8-89.6	80.3-95.2	85.4-99.1	90.6-100	NA
RT and old CT regimen	3-year COS	72.7	80.9	84.2	88.8	90.2	95.1
	95% CI	67.5-78.3	75.7-86.3	79.0-89.8	83.8-94.2	85.1-95.7	91.0-99.4
RT alone	3-year COS	75.7	83.2	86.7	92.1	89.7	93.2
	95% CI	68.5-83.6	75.9-91.2	79.2-94.9	85.6-99.1	80.3-100	84.6-100
	P	0.12	0.67	0.53	0.82	0.66	0.43

Abbreviations: RT, radiotherapy; CT: chemotherapy; COS, conditional overall survival; HR, hazard ratio; CI, confidence interval; NA, not available.

© 2019 Liu X et al. *JAMA Network Open*.

eTable 8. Three-year conditional overall survival probabilities stratified by treatment in high-risk patients

		Time since treatment					
Treatment		0 year	1 year	2 years	3 years	4 years	5 years
RT and new CT regimen	3-year COS	73.3	81.4	87.8	88.4	93.2	NA
	95% CI	68.3-78.7	75.3-87.9	80.9-95.4	78.9-99.1	84.3-100	NA
RT and old CT regimen	3-year COS	60.0	71.0	78.9	85.0	84.8	86.8
	95% CI	54.6-65.9	65.1-77.4	72.8-85.4	78.8-92.4	77.8-92.4	79.4-94.9
RT alone	3-year COS	46.5	66.1	75.4	86.1	80.5	NA
	95% CI	37.2-58.2	53.9-81.2	63.0-90.3	72.3-100	62.0-100	NA
RT and old CT regimen vs. RT and new CT regimen	HR for death	1.49	1.60	1.77	1.34	1.81	NA
	95% CI	1.13-1.95	1.07-2.39	0.94-3.33	0.54-3.56	0.41-8.01	NA
	P	0.004	0.02	0.07	0.53	0.43	NA
RT alone vs. RT and new CT regimen	HR for death	2.42	1.82	2.69	1.57	1.84	NA
	95% CI	1.73-3.39	1.05-3.17	1.23-5.90	0.46-5.28	0.30-11.2	NA
	P	<0.001	0.03	0.01	0.47	0.50	NA

Abbreviations: RT, radiotherapy; CT: chemotherapy; COS, conditional overall survival; HR, hazard ratio; CI, confidence interval; NA, not available.

© 2019 Liu X et al. *JAMA Network Open*.

© 2019 Liu X et al. *JAMA Network Open*.

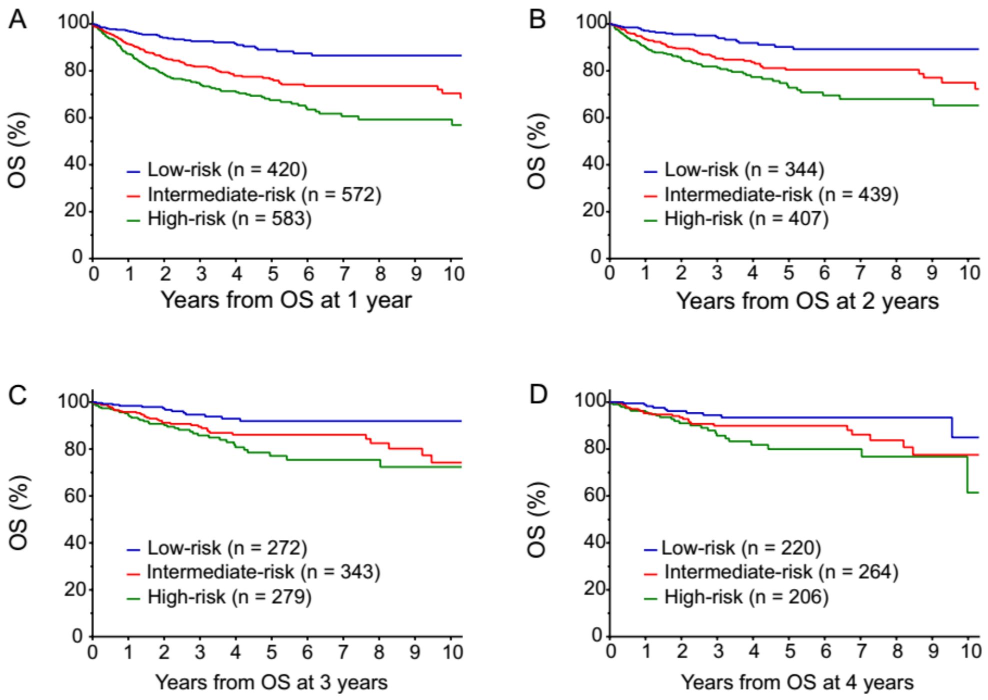

Supplemental Fig. 1

eFigure. Kaplan-Meier curves of overall survival stratified by risk groups

Kaplan-Meier curves of overall survival (OS) between low-risk, intermediate-risk and high-risk patients who were surviving at (A) 1 year, (B) 2 years, (C) 3 years and (D) 4 years.

© 2019 Liu X et al. *JAMA Network Open*.
